# Single-cell transcriptome reveals a novel mechanism of C-Kit^+^-liver sinusoidal endothelial cells in NASH

**DOI:** 10.1186/s13578-024-01215-7

**Published:** 2024-03-09

**Authors:** Hui-Yi Li, Yu-Xuan Gao, Jun-Cheng Wu, Jing-Ze Li, Seng-Wang Fu, Ming-Yi Xu

**Affiliations:** 1 Department of Gastroenterology, Shanghai East Hospital, Tongji University School of Medicine, No. 551, Pudong-South Road, Shanghai, 200120 China; 2 Department of Gastroenterology, Shanghai General Hospital, Shanghai Jiao Tong University School of Medicine, No. 100, Haining Rd, Shanghai, 200080 China; 3 Departments of Gastroenterology, The Third Affiliated Hospital of Soochow University, Changzhou, 213000 Jiangsu China; 4 Endoscopy Center, Shanghai East Hospital, Tongji University School of Medicine, Shanghai, 200120 China

**Keywords:** Non-alcoholic steatohepatitis (NASH), Liver sinusoidal endothelial cell (LSEC), Single-cell RNA sequencing (scRNA-seq), KIT proto-oncogene receptor tyrosine kinase (C-Kit), Mitophagy

## Abstract

**Aim:**

To understand how liver sinusoidal endothelial cells (LSECs) respond to nonalcoholic steatohepatitis (NASH).

**Methods:**

We profiled single-LSEC from livers of control and MCD-fed mice. The functions of *C-Kit*^+^-LSECs were determined using coculture and bone marrow transplantation (BMT) methods.

**Results:**

Three special clusters of single-LSEC were differentiated. *C-Kit*^+^-LSECs of cluster 0, *Msr1*^+^-LSECs of cluster 1 and *Bmp4*^+^*Selp*^+^-VECs of cluster 2 were revealed, and these cells with diverse ectopic expressions of genes participated in regulation of endothelial, fibrosis and lipid metabolism in NASH. The number of *C-Kit*^+^-primary LSECs isolated from MCD mice was lower than control mice. Immunofluorescence co-staining of CD31 and C-KIT showed *C-Kit*^+^-LSECs located in hepatic sinusoid were also reduced in NASH patients and MCD mice, compared to AIH patients and control mice respectively. Interestingly, lipotoxic hepatocytes/HSCs cocultured with *C-Kit*^+^-LSECs or the livers of MCD mice receipting of *C-Kit*^+^-BMCs (bone marrow cells) showed less steatosis, inflammation and fibrosis, higher expression of prolipolytic FXR and PPAR-α, lower expression of TNF-α and α-SMA*.* Furthermore, coculturing or BMT of *C-Kit*^+^-endothelial derived cells could increase the levels of hepatic mitochondrial LC3B, decrease the degree of mitochondrial damage and ROS production through activating *Pink1*-mediated mitophagy pathway in NASH.

**Conclusions:**

Hence, a novel transcriptomic view of LSECs was revealed to have heterogeneity and complexity in NASH. Importantly, a cluster of *C-Kit*^+^-LSECs was confirmed to recovery *Pink1*-related mitophagy and NASH progression.

**Supplementary Information:**

The online version contains supplementary material available at 10.1186/s13578-024-01215-7.

## Introduction

With the epidemiological burden of metabolic diseases, nonalcoholic fatty liver disease (NAFLD) has become the most common chronic liver disease in the world [1]. The pathogenesis of nonalcoholic steatohepatitis (NASH) is complex and controlled by the coordinated actions of liver cells, including hepatocytes (HCs), Kupffer cells (KCs), hepatic stellate cells (HSCs) and liver sinusoidal endothelial cells (LSECs). Recent a couple of single-cell RNA sequencing (scRNA-seq) studies have expounded the heterogeneity of liver cells and laid the foundation of the LSECs variability in NASH [2]. Xiong et al. proposed a concept of liver endothelial zonation and functional specialization in NASH [3]. Su et al. also identified an unanticipated aspect of 3 kind of chimeric NPCs (non-parenchymal cells) including LSECs chimeric HSCs/HCs/KCs in NAFLD mouse at the single-cell level [4]. Our scRNA-seq data locked up NPCs of NASH mice livers and screened out several LSEC subgroups with comparative obvious transition. LSECs comprise approximately 15–20% of the total number of liver cells, and line in the sinusoidal lumen of the liver sinusoids. However, during NAFLD development LSECs acquire a phenotype similar to vascular endothelial cells (VECs), actively promoting all pathophysiological aspects of NAFLD, including steatosis, inflammation and fibrosis [2]. LSEC dysfunction is critical for the progression to NASH while restoring LSEC homeostasis appears to be a promising approach to prevent NAFLD development and even reverse tissue damage [2]. Therefore, we conducted in-depth research on LSECs.

In this study, we screened different single-LSEC in methionine-choline deficient (MCD)-diet induced NASH and control-diet mice livers using scRNA-seq technology. The enriched gene signature of 3 subgroups of LSECs were demonstrated upon NASH injury. Then we focused on a subgroup of *C-Kit*^+^ (KIT proto-oncogene, receptor tyrosine kinase)-LSECs whose pathogenesis in NASH was unclear. Coculturing of *C-Kit*^+^-LSECs with HCs/HSCs, steatosis, inflammation, fibrosis and mitochondrial functions of the latter would be alleviated. Ultimately, implantation of *C-Kit*^+^-BMCs (bone marrow cells) into bone marrow transplantation (BMT) mice could improve their MCD-diet induced NASH and restoring the mitochondrial homeostasis.

## Materials and methods

### Human samples

Severe NASH patients (steatosis scores F3 and elevated serum ALT levels) and paired autoimmune hepatitis (AIH) patients (without NAFLD) were enrolled (each group n = 3). Biopsy liver tissues were collected. All enrolled patients provided written informed consent, and the study was approved by the ethics committee of Shanghai East Hospital.

### Mouse model

#### MCD-induced NASH mouse model

A total of 18 male C57BL/6 mice (8 weeks old, Shanghai SLAC Laboratory Animal Co. Ltd., Shanghai, China) were randomly assigned to control group (fed with chow diet, n = 9) and MCD group (fed with 40% carbohydrate, 10% fat, deficient in methionine and choline, n = 9). From 2 groups, 3 pairs were selected for scRNA-seq; another 3 pairs were prepared for primary LSECs (pLSECs) isolation and coculture experiment; last 3 pairs were performed for histological and immunofluorescence (IF) staining.

#### MCD-based BMT mouse model

Primary BMCs (pBMCs) were isolated from male C57BL/6 mice (Additional file 1). Preparation of donor BMCs was performed using the magnetic activated cell sorting (MACS) method (Miltenyi Biotec, Cologne, Germany). Pellets of pBMCs were suspended in MACS buffer, and 1 × 10^7^ total cells were incubated with 20 μL of C-KIT microbeads for 15 min in a refrigerator (2–4 °C). The LS column was washed with buffer and centrifuged to obtain *C-Kit*^−^-pBMCs. Remove the column from the separator and flush out the magnetically labeled cells with buffer to obtain *C-Kit*^+^-pBMCs. A total of 18 BMT recipient mice were first fed a MCD diet for 6 weeks. Then, recipient mice were lethally irradiated and subjected to BMT with *C-Kit*^+^- or *C-Kit*^−^-pBMCs [5]. All BMT mice were sacrificed after 2 weeks. The two resulting groups represented MCD_*C-Kit*^+^-BMC and MCD_*C-Kit*^−^-BMC (each group, n = 9). From 2 BMT groups, 3 pairs were selected for qPCR; another 3 pairs were chosen for western blot; last 3 pairs were performed for histological and IF staining.

#### Histological identification of the mouse model

Histological staining of H&E (hematoxylin–eosin), Masson (Masson trichrome), ORO (oil red O), and immunohistochemistry (IHC) of F4/80 (Abcam, Cambridge, MA, USA, Additional file 1: Table S1) were performed to identify the NASH model (Additional file 1). The animal study was approved by the Institutional Animal Care and Use Committee of Shanghai East Hospital.

### Primary cell isolation, cell line culture and treatment (see Additional file 1)

#### C-Kit^+^- and C-Kit^−^-pLSECs

The pLSECs were isolated from male C57BL/6 mice (Additional file 1), and then the MACS method (same as above) was used to prepared *C-Kit*^*+*^- and *C-Kit*^*−*^-pLSECs.

#### LSEC cell line (TMNK-1) transfection

For *C-Kit* silencing, short-hairpin RNA (shRNA) targeting human *C-Kit* (5′-CAACTGCTTATGGCTTAATTA-3′, sh-*C-Kit*) or a nonsense sequence (sh-NC) was inserted into the pLent-U6-shRNA-CMV-puro plasmid. For *C-Kit* overexpression (ov-*C-Kit*), full-length human *C-Kit* (ACCESSION: NM_001385292) was cloned and inserted into the pENTER plasmid, and an empty pENTER vector was used as a control (ov-NC). All vectors were purchased from Vigene Biosciences (Shandong, China). The transfection progress is shown in the Additional file 1.

### scRNA-seq analysis

#### Single-cell solution preparation

Liver tissues were digested in a Solo™ Tumor Dissociation Kit (Sinotech Genomics Co. Ltd., Shanghai, China, JZ-SC-58201). During enzymatic hydrolysis, HCs undergo breakage and apoptosis, and the assay process automatically filters out low-quality single cells; therefore, only NPCs were available in final single-cell analysis [6].

#### Single-cell transcriptome, library construction and sequencing

Cell concentration and viability were determined via a BD Rhapsody™ Scanner (BD Biosciences, San Jose, CA, USA). All procedures were performed with a BD Rhapsody cDNA Kit and BD Rhapsody Targeted mRNA & AbSeq Amplification Kit (BD Biosciences). All the libraries were sequenced in PE150 mode (pair-end for 150 bp reads) on the NovaSeq platform (Illumina, San Diego, CA, USA).

#### Dimensionality reduction, clustering and visualization

The Seurat v3.0 package was utilized for subsequent clustering analysis and visualization. Gene expression matrices for each sample were read and converted to Seurat objects. Cells with more than 5% mitochondrial unique molecular identifier (UMI) or less than 500 UMI or 200 genes were excluded from the downstream analysis. After log normalization based on the total cellular UMI count, a principal component analysis (PCA) was performed based on the top 2000 highly variable features after scaling the data with respect to UMI counts. Fifty principal components were used for clustering (nPC = 50). We then performed clustering at a resolution of 0.6 and visualized the data using either t-distributed stochastic neighbor embedding (t-SNE) or uniform manifold approximation and projection (UMAP). Feature plots, violin plots and heatmaps were used to visualize the expression of the indicated genes in each cluster.

#### Cell type annotation and differentially expressed gene (DEG) analysis

Specific marker genes for each cluster were calculated using the FindAllMarkers function with the Wilcoxon test [criteria: log2-fold change > 0.25, minimum (min.) percentage (pct) > 0.25]. To perform unbiased identification of cell types in filtered sample datasets and the combined dataset, we used the R package SingleR (v1.4.1), a computational framework that references bulk transcriptomes and helps annotate cell types for each cluster. The built-in Mouse RNAseq Data (MRD) in SingleR was used as the reference dataset. To identify DEGs in scRNA-seq, we used FindMarkers (Seurat R Package) with the Wilcoxon test and Bonferroni correction (criteria: min. pct > 0.25). Genes were regarded as upregulated or downregulated with a log2-fold change > 0.25 or < − 0.25 (adjusted *p-*value < 0.05). The ClusterProfiler package was utilized to detect enriched Kyoto Encyclopedia of Genes and Genome pathways (KEGG) or Gene Ontology (GO) biological functions from each set of DEGs. GO analysis included biological processes (BP), cell components (CC) and molecular functions (MF). We used the default parameters built into ClusterProfiler.

### Quantitative real-time PCR (qPCR)

QPCR was performed using a SYBR Green PCR Kit (Yeasen Biotech Co. Ltd., Shanghai, China) and ABI 7900HT Fast Real-Time PCR System (Applied Biosystems, Foster City, CA). The primers (Sangon Biotech Co. Ltd., Shanghai, China) used were listed in Additional file 1: Table S2. QPCR was repeated three times.

### Flow cytometry

Cells were mixed with appropriately diluted labeled antibodies at a 1:100 dilution (anti-C-KIT coupled with Alexa Fluor 647 Conjugate; Alexa Fluor 488 anti-CD31, Additional file 1: Table S1) incubated at 4 °C for 30 min. All samples were analyzed by a BD Accuri C6 flow cytometer (BD Biosciences), and FlowJo v10 software was used to analyze the data. This test was repeated three times.

### Cell coculture

Primary cell coculture was divided into 2 groups of pLSECs (*C-Kit*^+^ or *C-Kit*^−^) incubated in the upper chamber and treated with palmitic acid (PA). Cell line coculture was classified into 6 groups of TMNK-1 cells incubated in the upper chambers pretreated or transfected with BSA, PA, PA + sh-NC, PA + sh-*C-Kit,* PA + ov-NC and PA + ov-*C-Kit*.

Upper chamber cells were plated on polystyrene transwells (Corning Inc., Corning, NY, USA) with a 0.4 mm pore size at 3 × 10^5^ cells per well. Then, HCs [primary HCs (pHCs) or HepG2] or HSCs [primary HSCs (pHSCs) or LX2] were plated on 6-well dishes at 3 × 10^5^ cells per well. The upper cell-containing transwell was then placed into cell-containing 6-well dishes and cocultured for another 24 h. This process was repeated three times.

### Immunofluorescence (IF) assay

IF staining of cell or liver slides was achieved by incubation with anti-C-KIT, anti-CD31, anti-TNF (tumor necrosis factor)-α, anti-α-SMA (smooth muscle actin), anti-COX4 (cytochrome c oxidase subunit 4) and anti-LC3B (light chain 3B) at a 1:200 dilution (Abcam, Additional file 1: Table S1). DAPI was applied to show the nucleus. Representative images were captured via a TCS SP8 CARS fluorescence microscope (Leica Microsystems). Relative IF values were measured via ImageJ 1.8.0. This process was repeated three times.

### Western blot

Liver tissue lysates were homogenized in RIPA lysis and extraction buffer containing protease inhibitors (Millipore, Boston, USA). Total protein was quantified using the BCA Protein Assay Kit (GBCBIO, Guangzhou, China), and equal amounts of protein were separated by SDS-PAGE and transferred to PVDF membranes. Membranes were blocked with skimmed milk and incubated with primary and secondary antibodies (Additional file 1: Table S1). Membranes were developed using chemiluminescence reagents (Millipore), and the proteins were visualized on the ChemiDoc MP Imaging System (Bio-Rad). This process was repeated three times.

### Mitochondrial function test

#### Mitochondrial-SOX (mtSOX) IF staining

Mitochondrial ROS (reactive oxygen species) level was detected by mtSOX Red (Invitrogen, Carlsbad, USA) assay according to the manufacturer’s instructions. The samples were photographed via IF microscopy. The process were repeated three times.

#### Mitochondrial-Keima (mtKeima) IF staining

The transfection of mtKeima adenovirus was performed according to the manufacturer’s instructions (Hanbio Technology Co., Ltd. Shanghai, China). Cells grown on confocal dishes were transfected with mtKeima adenovirus at multiplicity of infection (MOI) of 50 for 6 h at 37 °C. The medium was then discarded and replaced with fresh medium containing the drugs. The cells were observed under confocal microscope. mtKeima is a pH-sensitive fluorescent protein, whose excitation spectrum shifts from 440 to 586 nm when mitochondria are delivered to acidic lysosomes, appearing as shift from green to red color. Mitophagy flux was monitored by evaluating the number of green and red puncta in each cell. The process were repeated three times.

### Statistical analysis

The statistical analyses used in each test was showed in Additional file 1: Table S3. Except scRNA-seq, data were presented as the mean ± standard deviation. A *p*-value < 0.05 was considered to indicate statistical significance.

## Results

### Cluster and spatial lobular location of single-LSEC was identified in MCD-induced NASH mice

**Fig. 1:**
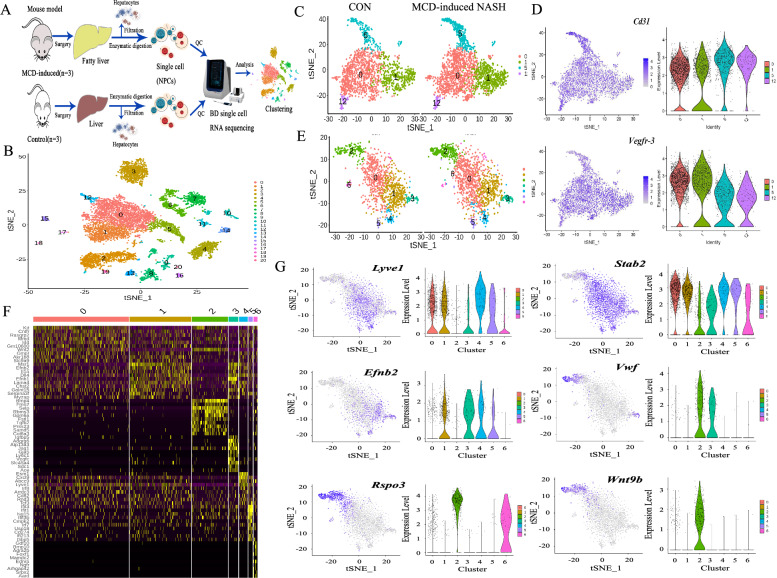
LSEC scRNA-seq analysis. **A** Liver single-cell isolation, detection and analysis workflow in MCD-induced NASH and control mice (each group n = 3). **B** t-SNE visualization of clusters based on the single-cell transcriptome. A total of 21 single-cell clusters (0–20) and 5 single-LSEC clusters (0, 1, 5, 12, 17) was shown. Each dot represented a single-cell, and each color represented a cluster. **C** t-SNE plots showed 4 clusters of single-LSEC population (cluster 0, 1, 5, 12) in control and NASH mice. **D** Paired t-SNE and violin plots showed the expression of marker genes of LSECs: *Cd31* and *Vegfr-3*. **E** t-SNE plots showed 7 new clusters of single-LSEC population (cluster 0–6) in control and NASH mice. **F** Heatmap of the top 10 representative DEGs of LSEC clusters (cluster 0–6). **G** Paired t-SNE and violin plots showed the expression of landmark DEGs: *Lyvel*, *Stab 2*, *Efnb2*, *Vwf*, *Rspo3* and *Wnt9b*

**Fig. 2:**
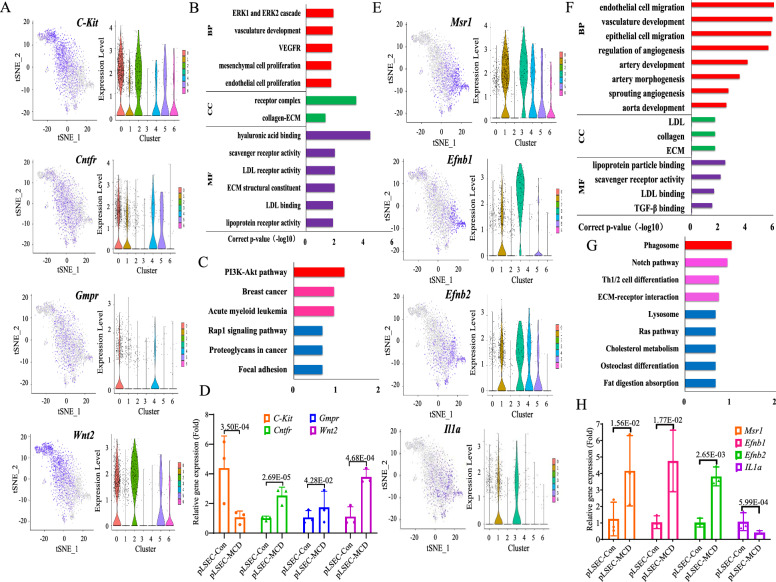
Transcriptomic scRNA-seq and PCR analysis of clusters 0 and 1. In cluster 0: **A** Paired t-SNE and violin plots showed the expression of *C-Kit*, *Cntfr*, *Gmpr* and *Wnt2*. **B**, **C** GO and KEGG analysis. **D** The mRNA levels of *C-Kit, Cntfr, Gmpr* and *Wnt2* in cluster 0 were examined by qPCR in pLSEC-Con and pLSEC-MCD group. In cluster 1: **E** Paired t-SNE and violin plots showed the expression of *Msr1, Efnb1*, *Efnb2* and *Il1a*. **F**, **G** GO and KEGG analysis. **H** The mRNA levels of *Msr1, Efnb1, Efnb2* and *IL1a* in cluster 1 by qPCR in pLSEC-Con and pLSEC-MCD group. In **D** and **H**, *p-*value indicated statistical significance compared to pLSEC-Con group

Elevated NASH activity scores (including steatosis, ballooning, lobular inflammatory cell infiltration, Additional file 1: Fig. S1A–E), more severe collagen accumulation (Additional file 1: Fig. S1B–F) and lipid deposition (Additional file 1: Fig. S1C–G), accompanied by more F4/80 staining (Additional file 1: Fig. S1D–H), were observed in MCD-fed mice than control mice. These findings demonstrated that MCD-fed mice developed to NASH. Liver NPCs from control or MCD mice were processed for scRNA-seq analysis (Fig. 1A). Clusters were annotated based on the gene expression of cell type-specific markers. A total of 21 single-cell clusters were revealed by a t-SNE plot (Fig. 1B). We focused on clusters of LSECs (cluster 0, 1, 5, 12, 17; Fig. 1B, C) characteristically expressing *Cd31* and *Vegfr* (vascular endothelial growth factor)*-3* (the EC marker genes; Fig. 1D). The cluster 0, 1 and 5 of LSECs were reclassified (cluster 12 and 17 with too few cells were omitted). Seven new clusters were identified between 2 groups (Fig. 1E). Additionally, LSECs of cluster 3, 4, 5 and 6, which had limited numbers, were omitted. A heatmap of the top 10 representative DEGs of all 7 clusters was shown (Fig. 1F). The spatial distribution of each cluster was determined based on the expression of well-known landmark genes using t-SNE and violin plots. *Lyve1* (lymphatic vessel endothelial receptor 1) and *Stab 2* (stabilin 2), known as LSEC markers, were expressed in most cells of cluster 0 and 1 (Fig. 1G). Interestingly, cells of cluster 1 also expressed periportal landmark such as *Efnb2* (recombinant ephrin b2, Fig. 1G). As a VEC marker, *Vwf* (von Willebrand factor) was uniquely expressed in cells of cluster 2 and 3 (Fig. 1G). *Rspo3* (recombinant R-spondin 3), *Wnt9b* and *Wnt2* are markers of central and pericentral VECs. *Rspo3* and *Wnt9b* were specifically expressed in cells of cluster 2 (Fig. 1G), whereas *Wnt2* was primarily expressed in cells of cluster 2 and 0 (Fig. 2A). The scRNA-seq technology differentiated 3 special clusters of LSECs in NASH disease. Collectively, cluster 0 and 1 were defined as LSECs, and cluster 2 was defined as VECs.

### A cluster of ***C-Kit***^+^-LSECs was identified in NASH

Cluster 0 was appraised based on the special expression of the top 10 representative DEGs (Table S4), including *C-Kit*, *Cntfr* (ciliary neurotrophic factor receptor), *Gmpr* (guanosine monophosphate reductase), *Wnt2* (Fig. 2A) and 6 other genes (*Akr1b8, Gm10600, Slc9a9, Id4, Mmd*, and *Rasgrp2*, Additional file 1: Fig. S2A–F and Additional file 1: Table S4). *C-Kit* was the most representative marker of cluster 0 since nearly 67% LSECs in cluster 0 were *C-Kit*^+^ [percentage fold change (pct-FC) was the 1st, Table S4]. Analysis of GO and KEGG, DEGs of these LSECs were associated to regulation of hyaluronic acid (HA), ERK1/2, VEGFR, mesenchymal cell proliferation and PI3K-AKT pathway (Fig. 2B, C). The pLSECs were isolated from control and MCD mice (pLSEC-Con and pLSEC-MCD group). Then, the top 4 representative DEGs of cluster 0 were examined by qPCR. Compared with pLSEC-Con group, *Cntfr*, *Gmpr* and *Wnt2* mRNA were upregulated, while *C-Kit* mRNA was downregulated in pLSEC-MCD group (*p* < 0.05, Fig. 2D). Therefore, a subgroup of *C-Kit*^+^-LSECs belonging to cluster 0 was identified, and they might participate in HA, ERK1/2, VEGFR and PI3K-AKT signaling transduction in NASH.

### Another cluster of ***Msr1***^+^-LSECs was found in NASH

The top 10 representative DEGs of cluster 1 were *Msr1* (macrophage scavenger receptor 1), *Efnb1* (ephrin b1), *Efnb2*, *Il1a* (interleukin 1a) (Fig. 2E and Additional file 1: Table S4) and 6 other genes (*Serpina3f, Lama4, Myzap, Dll4, Galnt15*, and *Chst2*, Additional file 1: Fig. S3A–F and Additional file 1: Table S4). *Msr1* was the most representative marker of cluster 1 since nearly 73% LSECs in cluster 1 were *Msr1*^+^ (pct-FC was the 1st, Additional file 1: Table S4). DEGs of these LSECs mainly regulated EC migration, vasculature development and angiogenesis (Fig. 2F, G). Compared with pLSEC-Con group, *Msr1* and *Efnb1/2* mRNA were upregulated, while *Il1a* was downregulated in pLSEC-MCD group (*p* < 0.05, Fig. 2H). Finally, another subgroup of *Msr1*^+^-LSECs classified under cluster 1 was found, and they appeared to participate in the regulation of endothelial functions in NASH.

### The third cluster of ***Bmp4***^+^***Selp***^+^-VECs was revealed in NASH

**Fig. 3:**
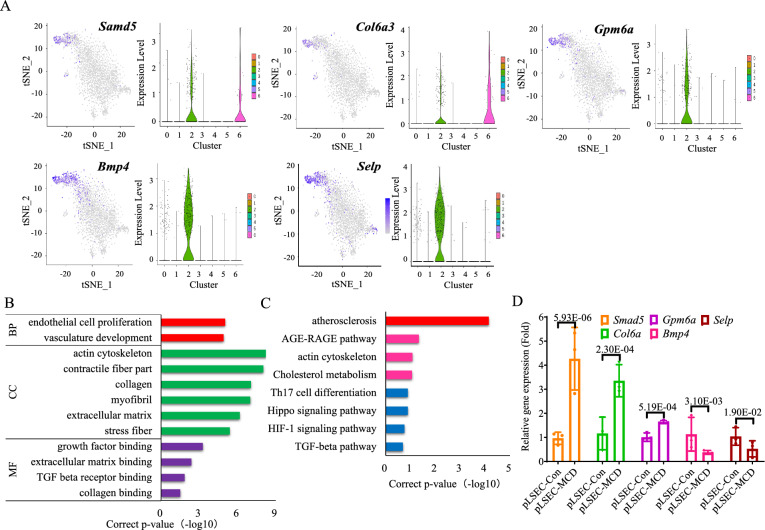
Transcriptomic scRNA-seq and PCR analysis of cluster 2. **A** Paired t-SNE and violin plots showing the expression of *Samd5*, *Col6a3, Gpm6a*, *Bmp4* and *Selp*. **B**, **C** GO and KEGG analysis. **D** The mRNA levels of *Samd5, Col6a, Gpm6a, Bmp4* and *Selp* in cluster 2 by qPCR in pLSEC-Con and pLSEC-MCD group. In **D**, *p-*value indicated statistical significance compared to pLSEC-Con group

The top 10 representative DEGs of cluster 2 included *Tgfb2*, *Fmo2*, *Prss23*, *Samd5* (sterile alpha motif domain 5), *Bmp4* (bone morphogenetic protein 4)*, Col6a3* (collagen 6α3)*, Gpm6a* (glycoprotein m6a)*, Fstl1, Selp* (selectin P, also LECAM3, CD62) and *Rbms3* (Fig. 3A and Additional file 1: Fig. S4A–E and Table S4). In cluster 2, 70–75% VECs were *Bmp4*^+^*Selp*^+^ (pct-FC was the 5th and 9th, Additional file 1: Table S4). DEGs of these VECs mainly regulated collagen, extracellular matrix (ECM), and atherosclerosis pathways (Fig. 3B, C). In cluster 2, all of the top 10 representative DEGs were examined by qPCR. Compared with pLSEC-Con group, *Samd5*, *Col6a3* and *Gpm6a* mRNA were upregulated, while *Bmp4* and *Selp* mRNA were downregulated in pLSEC-MCD group (*p* < 0.05, Fig. 3D). Therefore, a subgroup of hepatic *Bmp4*^+^*Selp*^+^-VECs from cluster 2 was revealed, and they were probably involved in the regulation of fibrosis and atherosclerosis in NASH.

### ***C-Kit***^+^-LSECs could improve NASH and mitophagy ***in vitro***

Among 3 clusters of LSECs differentiated by scRNA-seq, the cell number of *Bmp4*^+^*Selp*^+^-VECs was too limited and the mechanism of *Msr1* in NASH was already intensively clarified [7]. Then we focused on the *C-Kit*^+^-LSECs whose distinct roles in the pathogenesis of NASH should be fully elucidated.

**Fig. 4:**
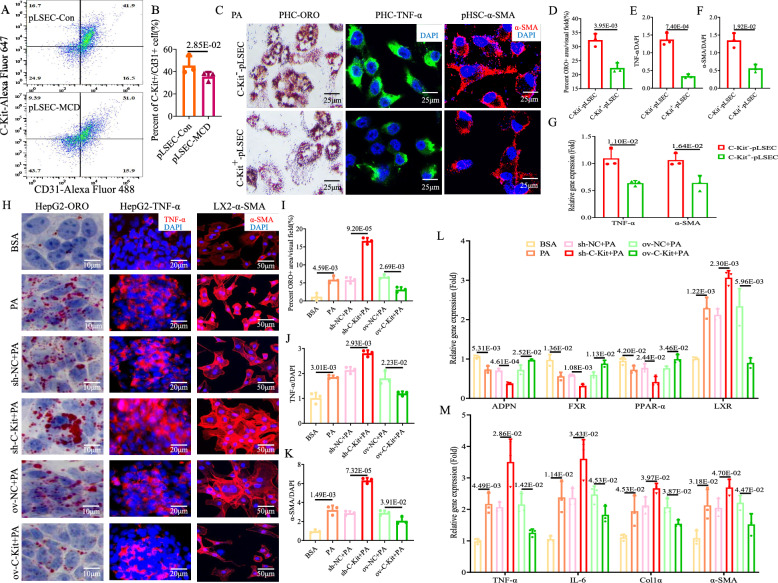
*C-Kit*^+^-LSECs alleviate NASH *in vitro*. **A**, **B** *CD31*^+^*C-Kit*^+^-pLSECs isolated from Con and MCD mice were detected by flow cytometry. **C**–**G** pHCs or pHSCs were cocultured with PA-treated *C-Kit*^+^- or *C-Kit*^−^-pLSECs. ORO staining **C** and calculation **D** of lipid droplets in pHCs (× 400). IF staining **C** and calculation **E** of TNF-α (green IF) in PHCs (× 400). IF staining **C** and calculation **F** of α-SMA (red IF) in pHSCs (× 400). DAPI (blue) was used for nuclear staining. (**G**) The mRNA of TNF-α (in pHCs) and α-SMA (in pHSCs) was detected by qPCR. (**H**-**M**) HepG2 or LX2 cells were cocultured with 6 groups of TMNK-1 cells (BSA, PA, sh-NC + PA, sh-*C-Kit* + PA, ov-NC + PA, ov-*C-Kit* + PA). ORO staining **H** and calculation **I** in HepG2 cells (× 1000). IF staining () and calculation **J** of TNF-α (red IF) in HepG2 cells (× 500). IF staining **H** and calculation **K** of α-SMA (red IF) in LX2 cells (× 200). DAPI (blue) was used for nuclear staining. The mRNA of **L** lipid metabolism genes (*APDN, FXR, PPAR-α, and LXR*) and **M** inflammation and fibrosis genes (*TNF-α, IL-6, Col1a,* and α*-SMA*) was examined by qPCR. The *p-*value indicated statistical significance compared to the pLSEC-Con, *C-Kit*^−^-pLSEC, BSA, sh-NC + PA or ov-NC + PA group

**Fig. 5:**
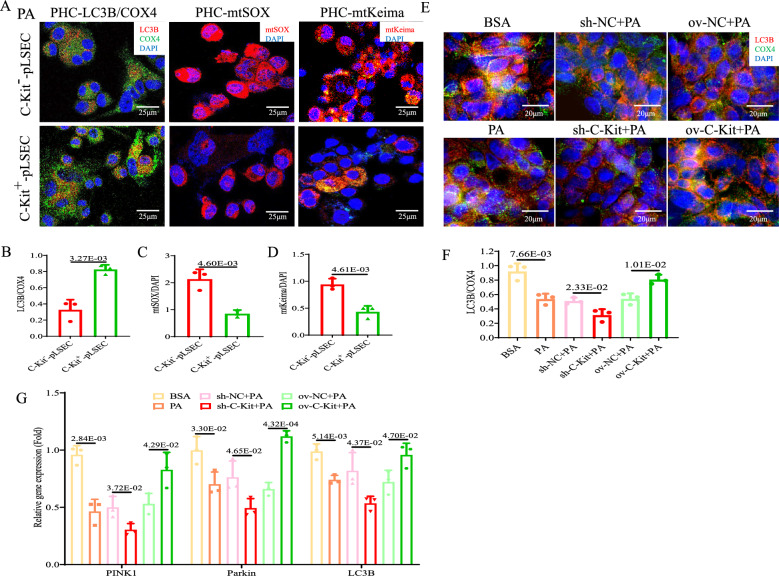
*C-Kit*^+^-LSECs improve the mitochondrial function of HCs *in vitro*. **A**–**D** pHCs were cocultured with PA-treated *C-Kit*^+^- or *C-Kit*^−^-pLSECs. IF staining and calculation of LC3B (red IF) and COX4 (green IF, A/B), mtSOX (red IF, A/C), and mtKeima (red IF, A/D) in pHCs (× 400). DAPI (blue) was used for nuclear staining. **E**–**G** HepG2 cells were cocultured with 6 groups of TMNK-1 cells. **E**, **F** IF staining and calculation of LC3B (red IF) and COX4 (green IF) in HepG2 cells (× 500). DAPI (blue) was used for nuclear staining. **G** The mRNA of mitophagy-related genes (*PINK1, Parkin, LC3B*) was examined by qPCR. The *p-*value indicated statistical significance compared to the *C-Kit*^−^-pLSEC, BSA, sh-NC + PA or ov-NC + PA group

Flow cytometric analysis revealed an obvious decreased percentage of *CD31*^+^*C-Kit*^+^-pLSECs derived from MCD mice compared to control mice (pLSEC-Con vs*.* -MCD group: 41.9% vs. 31.0%, *p* < 0.05, Fig. 4A, B). To explore the influence of *C-Kit*^+^-LSECs on peripheral cells, including HCs and HSCs, in a steatotic environment, we cocultured pHCs or pHSCs with *C-Kit*^+^- or *C-Kit*^−^-pLSECs in PA treatment. Significantly decreased lipid droplets were observed in pHCs cocultured with *C-Kit*^+^-pLSECs in comparison with *C-Kit*^−^-pLSECs (*C-Kit*^+^- vs*. C-Kit*^−^-pLSECs group: 0.65-fold, *p* < 0.05, Fig. 4C, D). TNF-α proteins (green IF) in pHCs and α-SMA proteins (red IF) in pHSCs were obviously reduced when cocultured with *C-Kit*^+^-pLSECs than *C-Kit*^−^-pLSECs (*C-Kit*^+^- vs*. C-Kit*^−^-pLSEC group: TNF-α was 0.23-fold, *p* < 0.05, Fig. 4C, E; α-SMA was 0.40-fold, *p* < 0.05, Fig. 4C, F). Also, mRNA of TNF-α and α-SMA were downregulated in cells cocultured with *C-Kit*^+^-pLSECs versus *C-Kit*^*−*^-pLSECs (Fig. 4G). Costaining of LC3B (autophagy proteins, red IF) and COX4 (mitochondrial proteins, green IF) shown orange IF. Interestingly, the manifestation of orange pHCs cocultured with *C-Kit*^+^-pLSECs was 3.36-fold higher than those with *C-Kit*^−^-pLSECs (*p* < 0.05, Fig. 5A, B). The mitochondrial ROS products (red IF of mtSOX, Fig. 5A, C) or damaged mitochondria (red IF of mtKeima, Fig. 5A, D) were 0.40-fold and 0.46-fold lower in pHCs cocultured with *C-Kit*^+^-pLSECs than with *C-Kit*^−^-pLSECs, respectively (*p* < 0.05).

Then, we cocultured HepG2/LX2 cells with 6 groups of TMNK-1 cells for additional validation of the effect of *C-Kit*. HepG2, cocultured with PA treated TMNK-1 cells, showed more lipid accumulation (Fig. 4H, I), downregulation of pro-lipolysis genes (*ADPN*: adiponectin, *FXR*: farnyl derivative X receptor, *PPAR-ɑ*: peroxisome proliferator-activated receptor-α, Fig. 4L) and upregulation of pro-lipogenesis genes (*LXR*: liver X receptor, Fig. 4L) compared to those cocultured with BSA treated TMNK-1 cells. Meanwhile, HepG2/LX2 cells cocultured with TMNK-1 cells of PA group displayed more TNF-α/α-SMA proteins (red IF, Fig. 4H, J and K), and upregulation of pro-inflammation and pro-fibrosis genes (TNF-α/IL-6, α-SMA/Col1a, Fig. 4M), compared to those cocultured with TMNK-1 cells of BSA group. Coculturing with TMNK-1 cells of *C-Kit* deficiency (sh-*C-Kit*) could aggravate the above lipotoxic damage to HepG2/LX2 cells, while coculturing with TMNK-1 cells of overexpressing *C-Kit* (*ov-C-Kit*) could reverse the above lipotoxic injury, compared to those with control cell groups (*p* < 0.05, Fig. 4H, M). Next, *Pink1* (PETN-induced putative kinase 1)*-*mediated mitophagy pathway was detected. After incubation with PA-treated TMNK-1 cells, HepG2 cells revealed significantly decreased LC3B/COX4 costaining (Fig. 5E, F) and lower mRNA levels of *Pink1*, *Parkin*, and *LC3B* (Fig. 5G) than with BSA-treated cells, suggesting that *Pink1*-mediated mitophagy in HCs was inhibited. Additionally, incubation with sh-*C-Kit* TMNK-1 cells could repress *Pink1*-related mitophagy pathway in HepG2 cells to a greater extent than those with sh-NC cells (*p* < 0.05, Fig. 5E, G). Conversely, incubation with ov-*C-Kit* TMNK-1 cells might significantly improve *Pink1*-related mitophagy in HepG2 cells compared to those with ov-NC cells (*p* < 0.05, Fig. 5E, G). Therefore, *C-Kit*^+^-LSECs would alleviate NASH by improving hepatic steatosis, inflammation, fibrosis and mitophagy *in vitro.*

### ***C-Kit***^+^-LSECs could alleviated NASH and recovery mitophagy ***in vivo***

**Fig. 6:**
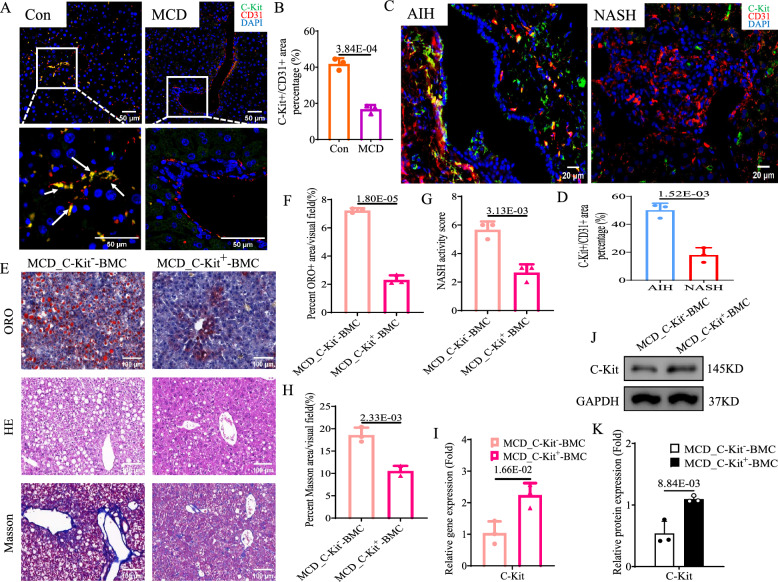
*C-Kit*^+^-LSECs were lack in NASH and BMT of *C-Kit*^+^-BMCs could protect against NASH *in vivo*. **A**, **B** Representative images and calculation of IF staining for C-KIT (green IF) and CD31 (red IF) in liver tissues of control and MCD mice (× 200). DAPI (blue) was used for nuclear staining. Arrows (yellow) indicated *C-Kit*^+^*CD31*^+^-LSECs. **C**, **D** Representative images and calculation of IF staining for C-KIT (green IF) and CD31 (red IF) in liver tissues of NASH and AIH patients (× 400). DAPI (blue) was used for nuclear staining. **C**–**K** We transplanted *C-Kit*^+^- or *C-Kit*^−^-BMCs into MCD mice. Images and calculation of ORO (E/F), H&E (E/G) and Masson (E/H) staining of liver tissues in 2 groups. **I** Hepatic *C-Kit* mRNA was examined by qPCR in 2 groups. **J**, **K** Hepatic protein levels of C-KIT were examined by western blot in 2 groups. The *p-*value indicated statistical significance compared to control mice, AIH patients or MCD_*C-Kit*^−^-BMC mice

**Fig. 7:**
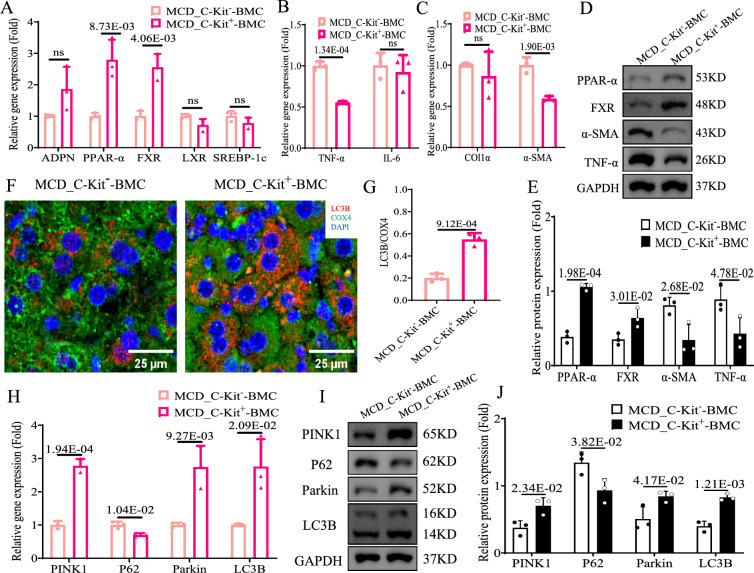
BMT of *C-Kit*^+^-BMCs might improve *Pink1*-mediated mitophagy and NASH *in vivo*. We transplanted *C-Kit*^+^- or *C-Kit*^−^-BMCs into MCD mice. **A**–**C** Hepatic mRNA was examined by qPCR in 2 groups: **A** *APDN, PPAR-α, FXR, LXR* and *SREBP-1c*; **B** *TNF-α* and *IL-6*; **C** *Col1a* and α*-SMA*. **D**, **E** Hepatic protein levels of PPAR-α, FXR, α-SMA, and TNF-α were examined by western blot in 2 groups. **F**, **G** IF staining and calculation of COX-4 (green IF) and LC3B (red IF) in liver tissues of 2 groups (× 400). DAPI (blue) was used for nuclear staining. The liver mRNA **H** and protein **I**, **J** levels of *Pink1*-mediated mitophagy pathway were examined by qPCR and western blot in 2 groups. The *p-*value indicated statistical significance compared to MCD_*C-Kit*^−^-BMC mice

Lower percentage of *C-Kit*^+^*CD31*^+^ cell (showed orange IF staining) was seen in hepatic sinusoids of MCD mice than control mice (MCD vs*.* control group: 0.37-fold, *p* < 0.05, Fig. 6A, B). To determine the state of *C-Kit*^+^-LSECs in real-world NASH, we also checked the percentage of *C-Kit*^+^*CD31*^+^ cells in severe NASH and AIH patients (as control). The livers of AIH patients contained abundant *C-Kit*^+^*CD31*^+^ cells, but the livers of severe NASH patients showed rare *C-Kit*^+^*CD31*^+^ cells in hepatic sinusoids (NASH vs. AIH group: 0.31-fold, *p* < 0.05, Fig. 6C, D). To determine the remedy function of *C-Kit*^+^-LSECs in NASH *in vivo*, we transplanted *C-Kit*^+^- or *C-Kit*^−^-BMCs into MCD-induced NASH mice (representing MCD_*C-Kit*^+^-BMC and MCD_*C-Kit*^−^-BMC group). Relative to MCD_*C-Kit*^−^-BMC mice, hepatic steatosis (Fig. 6E, F), lobular inflammation (Fig. 6E, G) and fibrosis (Fig. 6E, H) were significantly alleviated in MCD_*C-Kit*^+^-BMC mice (*p* < 0.05). The mRNA and protein levels of C-Kit (Fig. 6I, G and K), PPAR-α and FXR (Fig. 7A, D and E) were consistently higher, while TNF-α (Fig. 7B, D and E) and α-SMA (Fig. 7C, D and E) were accordantly lower in MCD_*C-Kit*^+^-BMC mice than in MCD_*C-Kit*^−^-BMC mice (*p* < 0.05). Then, the transition of *Pink1*-mediated mitophagy was examined *in vivo*. In MCD_*C-Kit*^−^-BMC mice, the IF value of hepatic costaining of LC3B/COX4 was increased by 2.89-fold compared with that in MCD_*C-Kit*^−^-BMC mice (*p* < 0.05, Fig. 7F, G). Compared to those in MCD_*C-Kit*^−^-BMC mice, the mRNA and protein levels of Pink1, Parkin and LC3B were significantly increased, and those of p62 were obviously decreased in MCD_*C-Kit*^+^-BMC mice (Fig. 7H, I and J). These results suggested that BMT of *C-Kit*^+^-BMCs could ameliorate *Pink1*-mediated mitophagy and MCD-induced NASH *in vivo*.

## Discussion

NAFLD is the most frequent chronic liver disease worldwide, representing 25% of the population [8]. NASH, a liver condition characterized by steatosis, inflammation and/or fibrosis, can progress to cirrhosis. NAFLD (foretastes to increase 21%) and resulting NASH (increase 63%) are highly prevalent in the United States, where they are a growing cause of cirrhosis (increase 168%) and hepatocellular carcinoma (HCC, increase 137%), and they are expected to become the most common cause of liver transplants by 2030 [9]. Despite this substantial health and economic burden, therapeutic options for NAFLD/NASH remains limited owing to the lack of a deep understanding of the cellular and molecular mechanisms. LSECs represent 40% of the NPC population in the human liver [10]. Emerging literature implicates LSECs in the pathogenesis and progression of NAFLD/NASH [11]. During the evolution of NASH, LSECs undergo phenotypic changes as capillarization consisting of reduced size and number of fenestrae and deposition of basement membrane on the abluminal side [12]. Then LSECs secrete in response to lipotoxic stress and chemokines/cytokine stimulation who enhance the trafficking of immune cells to the liver [13]. Understanding when and how LSECs respond to the lipotoxic microenvironment of NASH is currently unclear.

A better understanding of the roles of different cell types in the process is critical for prevention and management of NASH. Recent technical advances in single-cell analysis have characterized distinct sub-populations of the LSECs, defined their gene expression profile, and broadened our understanding of their mechanistic role in NAFLD/NASH [3, 4, 6]. Our work also generated a single-cell signature that could represent a damaged LSEC population in MCD-induced NASH mice. Independent scRNA-seq analysis of single-LSEC filtrated out 3 meaningful clusters in MCD and control mice livers. (1) LSECs of cluster 0 specifically expressed *C-Kit*, *Cntfr*, *Gmpr*, *Wnt2* and so on. Interestingly, 67% cells of cluster 0 were *C-Kit*^+^, and *C-Kit* mRNA was downregulated in pLSECs of MCD mice (to be discussed later). Our data also showed 49%, 27%, 59% cells of cluster 0 were *Cntfr*^+^, *Gmpr*^+^*, Wnt2*^+^, and their mRNAs were upregulated in pLSCEs of MCD mice. There are no researches elaborate the role of *Cntfr* and *Gmpr* in LSECs. Ding et al. clarified that *Wnt2* would re-establish vascular niche in the liver sinusoids and restore hepatovascular regeneration as a LSEC-derived angiocrine factor [14]. Thus, our findings partly reflected that *Wnt2*^+^-LSECs were stimulated in NASH occurrence. (2) LSECs of cluster 1 universally expressed *Msr1, Efnb1/2*, *Il1a*, and so on. Approximate 73% cells of cluster 1 were *Msr1*^+^, and *Msr1* mRNA was upregulated in pLSECs of MCD mice. Govaere et al. found MSR1 expression was correlated with the degree of steatosis and steatohepatitis in NASH patients, while global knockout of *Msr1* played a protective role with decreased macrophages, less inflammation and improved lipid metabolism in NASH mice [7]. Our data also identified *Msr1*^+^-LSECs were stimulated in the progression of NASH. Then in cluster 1, our data showed 41%, 47%, 34% cells were *Efnb1*^+^, *Efnb2*^+^, *Il1a*^+^, and mRNAs of *Efnb1/2* were upregulated, while *Il1a* was downregulated in pLSCEs of MCD mice. There are no researches reveal the role of *Efnb1/2* and *Il1a* in LSECs. (3) Hepatic VECs of cluster 2 characteristically expressed *Samd5*, *Col6a3*, *Gpm6a*, *Bmp4* and *Selp*. In cluster 2, 70–75% VECs were *Bmp4*^+^*Selp*^+^, their mRNAs were downregulated in pLSECs of MCD mice. Gage et al. reported that *Bmp4*^+^-VECs engrafted into the mouse liver could significantly promote proliferation and mature to functional LSECs [15]. While the effect of *Selp*^+^-LSECs is still unknown, then the mechanism of *Bmp4*^+^*Selp*^+^-VECs in NASH livers need further explore. In addition in cluster 2, our data showed 30%, 25%, 46% cells were *Samd5*^+^, *Col6a3*^+^, *Gpm6a*^+^, and mRNAs of *Samd5*, *Col6a3*, *Gpm6a* were upregulated in pLSCEs of MCD mice. Nevertheless the role of these 3 genes in LSECs is unclear. The mechanisms of these subgroups of LSECs and their genomic feature would be interesting to explore. The mechanism of heterogeneity of the LSEC subpopulations, which were identified by the recent scRNA-seq technique, maybe combination of transcriptional regulators, epigenetic mechanisms, or microenvironmental factors [16]. Future work will have to address which transcription factors are responsible for LSEC heterogeneity.

After MCD-induced NASH injury, we observed a key cluster of *C-Kit*^+^-LSECs with a changed phenotype. Usually, bone marrow-derived endothelial progenitor cells (EPCs) are *C-Kit*^+^ [17]. During EPC differentiation into mature ECs, circulating EPCs gradually lose the expression of *C-Kit* and then express mature EC markers (*CD31*, etc.) [18]. Subsequently, a group of hemangioblast-like cells were found in adult tissues as well, suggesting that such embryonic cells may reappear during the onset of disease [19]. Deng et al. first performed scRNA-seq analysis of the whole aorta and revealed that *C-Kit*^+^-cells were a major source of ECs in atheroprone regions of the aorta and transplant arteriosclerotic lesions [20]. Crosby et al. innovatively identified a *C-Kit*^+^-cell population with stem cell characteristics located in the hepatic portal area of adult cirrhotic and normal livers, and some cells were *CD31*^+^ [21]. We hypothesized that *C-Kit*^+^-LSECs were also hemangioblast-like cells, whose functions were unclear and lacked previous proof in NASH.

Recently, Duan et al. reported that LSEC senescence could promote steatosis by inactivating pericentral endothelium-derived *C-Kit*; while infusing *C-Kit*^+^-LSECs into aged NASH mice could counteract senescence and steatosis [22]. We first proved that *C-Kit*^+^-LSECs were markedly decreased in NASH by flow cytometry of pLSECs and IF staining of liver tissues (both in human and in mice). Compared to *C-Kit*^−^-LSECs, we secondly clarified that *C-Kit*^+^-LSECs had the abilities to reverse steatosis, inflammation and fibrosis of NASH; while upregulate prolipolytic FXR/PPAR-α, downregulate proinflammational TNF-α and profibtotic α-SMA *in vitro* and *in vivo*. However, the role of *C-Kit*^+^-LSEC derived factors in the evolution of NASH during liver injury is an area ripe for further investigation.

Damaged mitochondria release mitochondrial ROS and DNA into the cytosol, which acts as danger signals resulting in the hyperactivation of inflammatory signalling pathways [23]. Korski et al. proposed that oxidative stress, mitochondrial dysfunction and cellular energy imbalance could arrest early proliferation of *C-Kit*^+^-CPCs (cardiac progenitor cells) [24]; Rahman et al. also found pharmacologically inhibiting of mitochondrial fragmentation could retain the undifferentiated state of *C-Kit*^+^-CPCs [25]. Mitochondrial dysfunction is already assumed involved in the pathology of NASH with diverse mechanisms especially mitophagy (a selective autophagy eliminating damaged mitochondria to maintain mitochondrial homeostasis) [26]. *Pink1*-dependent mitophagy is a well-known signalling cascade that recognizes cargo through the polyubiquitination of mitochondrial proteins and recruits the autophagic machinery [27]. Gao et al. proved inhibiting *Pink1*-mediated mitophagy could promote pyroptosis in steatotic HCs in NASH [28]. We previously elucidated that inhibition of *Pink1*-mediated mitophagy would enhance HSC activation and accelerate liver fibrosis in NASH [29]. In this research, we unveiled that steatotic HCs, which cocultured with *C-Kit*^+^-LSECs *in vitro* or transplanted with *C-Kit*^+^-BMCs *in vivo*, exhibited more mitochondrial LC3B proteins, or less mitochondrial damage (mtKeima) and ROS products (mtSOX) through stimulating *Pink1*-mediated mitophagy pathway. Interestingly, the above-mentioned molecular pathways converge into a common point: mitochondrial dysfunction, which critically determines the activity of the oxidative phosphorylation cascade and is associated with early proapoptotic events and defects in fatty acid oxidation [30]. Therefore, *C-Kit*^+^-LSECs participate in alleviation of NASH by improving hepatic mitochondrial function, steatohepatitis and fibrosis*. C-Kit* mediated *Pink1*-related mitophagy maybe one of the complementary mechanisms underlying mitochondrial adaptation in NASH.

In summary, a novel transcriptomic view of LSECs was revealed to have heterogeneity and complexity in NASH by scRNA-seq analysis. Three subgroups of LSECs were summarized in detail based on DEGs and GO and KEGG enrichment in NASH. Importantly, a cluster of *C-Kit*^+^-LSECs was confirmed to stimulate *Pink1*-related mitophagy and recovery NASH progression.

### Supplementary Information

**Additional file 1: Table S1.** Antibodies used in IF/IHC, Flow Cytometry and western blot. **Table S2.** Primers used in qPCR. **Table S3.** Statistical analyses used in the methods. **Table S4.** The top 10 representative DEGs of cluster 0, 1, 2 from LSECs. **Figure S1.** Histological examination of mice model. **Figure S2.** Expression of 6 DEGs of cluster 0. **Figure S3.** Expression of 6 DEGs of cluster 1. **Figure S4.** Expression of 5 DEGs of cluster 2.

## Data Availability

The datasets generated during and/or analyzed during the current study are available from the corresponding author on reasonable request. Sequence data that support the findings of this study is available through GEO database (number GSE225786).
