# Supplementary material for: Single-cell transcriptome reveals a novel mechanism of C-Kit+-liver sinusoidal endothelial cells in NASH
Source: Cell Biosci. 2024 Mar 9;14:31. doi: 10.1186/s13578-024-01215-7 (PMC10925010; doi:10.1186/s13578-024-01215-7)
Supplement: Supplementary file 1 — Additional file 1: Table S1. Antibodies used in IF/IHC, Flow Cytometry and western blot. Table S2. Primers used in qPCR. Table S3. Statistical analyses used in the methods. Table S4. The top 10 representative DEGs of cluster 0, 1, 2 from LSECs. Figure S1. Histological examination of mice model. Figure S2. Expression of 6 DEGs of cluster 0. Figure S3. Expression of 6 DEGs of cluster 1. Figure S4. Expression of 5 DEGs of cluster 2. [file 13578_2024_1215_MOESM1_ESM.docx]

**ADDITIONAL MATERIAL**

**Single‑cell transcriptome reveals a novel mechanism of C-Kit^+^-liver sinusoidal endothelial cells in NASH**

Hui-Yi Li^1#^, Yu-Xuan Gao^1#^, Jun-Cheng Wu^3#^, Jing-Ze Li^4^, Seng-Wang Fu^2*^, Ming-Yi Xu^1*^

1. Department of Gastroenterology, Shanghai East Hospital, Tongji University School of Medicine, Shanghai, 200092, China
2. Department of Gastroenterology, Shanghai General Hospital, Shanghai Jiao Tong University School of Medicine, Shanghai, 200080, China
3. Departments of Gastroenterology, The Third Affiliated Hospital of Soochow University, Changzhou, Jiangsu Province, 213000, China
4. Endoscopy Center, Shanghai East Hospital, Tongji University School of Medicine, Shanghai, 200092, China

**ABBREVIATION**

nonalcoholic fatty liver disease (NAFLD)

nonalcoholic steatohepatitis (NASH)

hepatocytes (HCs)

kupffer cells (KCs)

hepatic stellate cells (HSCs)

liver sinusoidal endothelial cells (LSECs)

single-cell RNA sequencing (scRNA-seq)

endothelial cells (ECs)

nonparenchymal cells (NPCs)

methionine-choline deficient diet (MCD)

KIT proto-oncogene, receptor tyrosine kinase (C-Kit)

primary bone marrow cells (pBMCs)

magnetic activated cell sorting (MACS)

hematoxylin-eosin (H&E)

oil red O (ORO)

Masson trichrome (Masson)

immunohistochemistry (IHC)

primary HCs (pHCs)

primary LSECs (pLSECs)

primary HSCs (pHSCs)

short-hairpin RNA (shRNA)

1. *Kit* overexpression (ov-*C-Kit*)

unique molecular identifiers (UMI)

principal component analysis (PCA)

t-distributed Stochastic Neighbor Embedding (t-SNE)

uniform manifold approximation and projection (UMAP)

differential expressed gene (DEG)

minimum (min.)

percentage (pct)

Kyoto Encyclopedia of Genes and Genome pathways (KEGG)

Gene Ontology (GO)

biological processes (BP)

cell components (CC)

molecular functions (MF)

quantitative real-time PCR (qPCR)

palmitic acid (PA)

primary HCs (pHCs)

primary HSCs (pHSCs)

immunofluorescence (IF)

mitochondrial-SOX (mtSOX)

mitochondrial-Keima (mtKeima)

tumor necrosis factor -α (TNF-α)

smooth muscle actin-α (α-SMA)

cytochrome c oxidase subunit 4 (COX4)

light chain 3B (LC3B)

reactive oxygen species (ROS)

vascular endothelial growth factor (Vegfr)

transforming growth factor-β1 (TGF-β1)

lymphatic vessel endothelial receptor 1 (*Lyve1*)

stabilin 2 (*Stab2*)

recombinant ephrin b2 (*Efnb2*)

vascular ECs (VECs)

von Willebrand factor (*Vwf*)

recombinant R-spondin 3 (*Rspo3*)

ciliary neurotrophic factor receptor (*Cntfr*)

guanosine monophosphate reductase (*Gmpr*)

percentage fold change (pct-FC)

hyaluronic acid (HA)

macrophage scavenger receptor 1 (*Msr1*)

ephrin b (*Efnb*)

interleukin 1a (*Il1a*)

sterile alpha motif domain 5 (*Samd5*)

bone morphogenetic protein 4 (*Bmp4*)

collagen 6α3 (*Col6a3*)

glycoprotein m6a (*Gpm6a*)

selectin P (*Selp*)

extracellular matrix (ECM)

adiponectin (ADPN)

Farnyl derivative X receptor (FXR)

peroxisome proliferator-activated receptor-α (PPAR-α)

liver X receptor (LXR)

phosphatase and tensin induced putative kinase 1 (*Pink1*)

endothelial progenitor cell (EPC)

free fatty acid (FFA)

cardiac progenitor cell (CPC)

**SUPPLEMENTAL METHODS**

**Histological identification of mice model**

Mouse liver tissue sections were prepared and stained with H&E (hematoxylin-eosin), Masson (Masson trichrome) and ORO (oil red O). Immunohistochemistry (IHC) of liver macrophages were detected using anti-mouse F4/80 antibody. F4/80 positive cells were counted. Steatohepatitis, lipid droplets, fibrosis and inflammatory infiltration of liver were observed by light microscopy (Leica Microsystems, Wetzlar, Germany). Images were analyzed using Image J 1.8.0 software (National Institutes of Health, USA). A diagnosis of NASH activity score was evaluated by the pathologist according to the published criteria ^[1]^, which was determined by steatosis, inflammation and balloon swelling.

**Mouse primary cells isolation and culture**

**Primary LSECs (pLSECs)**

pLSECs were isolated from C57BL/6 male mice (standard diet as control group, MCD diet as MCD group for 6-weeks) as previously described ^[2]^. Mouse livers were perfused through the inferior vena cava with warm collagenase (Roche, Basel, Switzerland), and the portal vein was sectioned. The liver was removed and mechanically dissociated. Cells were isolated by centrifugation through Percoll gradients (Yeasen Biotech Co. Ltd.) to obtain pLSECs. The pLSECs were cultured in ECM media (ScienCell, Carlsbad, CA, USA) supplemented with 5% fetal bovine serum (FBS; Invitrogen, Carlsbad, CA, USA), antibiotics and antimycotics (Sigma-Aldrich, St. Louis, MO, USA).

**Primary HCs (pHCs) and HSCs (pHSCs)**

Isolation of PHCs and pHSCs were isolated from C57BL/6 mice as our previously study ^[2]^. pHSCs were isolated using a gradient centrifugation method. After perfusing the livers with collagenase and pronase (Roche), the pHSCs were isolated by Nycodenz density gradient (Sigma-Aldrich) centrifugation. The cells were subsequently cultured in DMEM (HyClone South Logan, UT, USA) supplemented with 10% FBS and antibiotics. PHCs were isolated using a two-step collagenase digestion method. PHCs were cultured with M199 medium (Gibco) supplemented with 10% FBS and antibiotics. After 1-7 days in culture, the cells were harvested for the subsequent experiments.

**Primary BMCs (pBMCs)**

Isolation of pBMCs was referred to previously reported method ^[3]^. The 6-weeks C57BL/6 male mice (n=5) were euthanized utilizing CO_2_. Dissect the skin of bilateral lower limbs, isolate femur and tibia completely. Cut both ends of the tibia or femur, rinse the bone marrow cavity with buffer through a syringe. Filter the flush solution and centrifuge at 600G for 5 min (4℃) to obtain the resuspension of pBMCs. The pBMCs were subsequently cultured in DMEM supplemented with 10% FBS and antibiotics. Determine cell concentration with automated cell counter. Typical yield can range from 1×10^7^ to 1.2×10^7^ progenitor cells/mouse.

**Cell line**

TMNK-1 (human LSEC), HepG2 (human hepatoma cell) and LX2 (human HSC) were cultured in DMEM containing 10% FBS and antibiotics. Before *in vitro* experiment, cells were first incubated in serum-free media overnight. To induce a lipotoxic environment, TMNK-1 cells were pretreated with palmitic acid (PA, 200 μM; Sigma-Aldrich) or 3% BSA as the vehicle control for 24 hours. LX2 cells were pretreated with transforming growth factor-β1 (TGF-β1, 10 ng/ml; Sigma-Aldrich) for 24 hours.

**Cell line transfection**

Logarithmic growth of TMNK-1 cells was selected and seeded into 6-well plates at a density of 1×10^5^ cells /mL with 2 ml of DMEM supplemented with 10% FBS, and cultured at 37℃ with 5% CO_2_. Transfection was conducted at 40-60% confluence. Lipofectamine 3000 (Invitrogen) was used for transfection of plasmids into TMNK-1 cells according to the manufacturer’s protocol. Transduction effectiveness was investigated following 48 hours through reverse transcription quantitative polymerase chain reaction (RT-qPCR).

**ScRNA-seq analysis**

**Single-cell solution preparation**

Liver tissues were cut into about 1-2mm^3^ pieces and digested in Solo^TM^ Tumor Dissociation Kit at 37°C for 30-60 minutes. Then we stopped enzymatic digestion with excess RPMI-1640 medium, and filtered cell with 40 μm cell strainer. NPCs were available in the final single-cell analysis. Single cell solution was kept on ice before loading to BD Rhapsody cartridge for single cell transcriptome capture (Sinotech Genomics Co. Ltd., Shanghai, China). Samples from MCD induced NASH and control groups were pooled separately for scRNA-seq analysis.

**Single-cell transcriptome, library construction and sequencing**

Cells were loaded in one BD Rhapsody microwell cartridge and lysed with lysis buffer, and the cell capture beads were retrieved and washed prior to performing reverse transcription. The microbead-captured single-cell transcriptome was converted into a cDNA library containing cell labels and UMI information.

**Sequencing data processing**

Raw sequencing reads of the cDNA library were processed through the BD Rhapsody Whole Transcriptome Assay Analysis Pipeline (v1.8), which included filtering by read quality, annotating reads, annotating molecules, determining putative cells and generating a single-cell expression matrix. Among all the output files, the matrix of UMI counts for each gene per cell was used for downstream analysis. Genome Reference Consortium Mouse Build 38 (GRCm38) was used as a reference for the BD pipeline.

**Reference**

1. Younossi ZM, Loomba R, Anstee QM, et al. Diagnostic modalities for nonalcoholic fatty liver disease, nonalcoholic steatohepatitis, and associated fibrosis. Hepatology. 2018; 68: 349-360.
2. Tao L, Ma W, Wu L, et al. Glial cell line-derived neurotrophic factor (GDNF) mediates hepatic stellate cell activation via ALK5/Smad signalling. Gut. 2019; 68: 2214-2227.
3. Bi Y, Guo X, Zhang M, et al. Bone marrow derived-mesenchymal stem cell improves diabetes-associated fatty liver via mitochondria transformation in mice. Stem Cell Res Ther. 2021;12: 602.

**Additional Material**

**Table S1 Antibodies used in IF/IHC, Flow Cytometry and western blot**

| **Antibody** | **Species** | **Manufacturer** | **Catalog #** | **Application** | **Concentration (mg/ml)** |
| --- | --- | --- | --- | --- | --- |
| F4/80 | mouse | Abcam | ab6640 | IHC | 1 |
| C-Kit | mouse/human | Affinity | BF8286 | Flow cytometry, IF, western blot | 0.5 |
| CD31 | human | Affinity | AF0077 | Flow Cytometry, IF | 1 |
| Alexa Fluor 488 anti-mouse CD31 | mouse | BioLegend | 102414 | Flow Cytometry, IF | 0.5 |
| Alexa Fluor 647 Conjugate | mouse | Cell Signaling Technology | 4418S | Flow Cytometry | 2 |
| α-SMA | mouse | Abcam | ab124964 | IF | 0.147 |
| TNF-α | mouse | Abcam | ab183218 | IF | 0.476 |
| LC3B | mouse | Abcam | ab63817 | IF, western blot | 0.5 |
| COX4 | mouse | Abcam | ab33985 | IF | 1 |
| Alexa Fluor 488 AffiniPure Donkey Anti-Rabbit IgG IgG(H+L) | mouse | Yeasen | 34106ES60 | IF | 0.75 |
| Alexa Fluor 594 AffiniPure Donkey Anti-Rabbit IgG (H+L) | Rabbit | Yeasen | 34212ES60 | IF | 0.75 |
| PPAR-α | mouse | Proteintech | 66826-1-Ig | western blot | 1 |
| FXR | mouse | Proteintech | 25055-1-AP | western blot | 0.9 |
| α-SMA | mouse | Proteintech | 14395-1-AP | western blot | 0.5 |
| TNF-α | mouse | Proteintech | 60291-1-Ig | western blot | 2 |
| PNIK1 | mouse | Affinity | DF7742 | western blot | 1 |
| P62 | mouse | Proteintech | 66184-1-Ig | western blot | 2 |
| Parkin | mouse | Affinity | AF0235 | western blot | 1 |
| Rabbi anti-GAPDH | rabbit | Goodhere | AB-P-R 001 | western blot | 1 |
| HRP Conjugated AffiniPure Goat Anti-Rabbit IgG (H+L) | rabbit | Boster | BA1054 | western blot | 1 |

**Table S2 Primers used in qPCR**

| **Gene** | **species** | **GenBank Accession** | **Exon Location** | **Primer Sequence (5'-3')** |
| --- | --- | --- | --- | --- |
| *C-Kit* | human | NM_000222 | 1174-1289  bp | F: GCACCGAAGGAGGCACTTAC R: AATCCTGCTGCCACACATTG |
| *Cntfr* | human | NM_001842 | 256-489  bp | F: CTGGGCTCTGACGTGACAC R: GTGGAAGCAGGCGTAGAGG |
| *Gmpr* | human | NM_006877 | 655-761  bp | F: GAGTGCCGTCATTGAGTGTG R: TCCGTATGACCCGAAAACAT |
| *Wnt2* | human | NM_003391 | 1-152  bp | F: CCGAGGTCAACTCTTCATGGT R: CCTGGCACATTATCGCACAT |
| *Msr1* | human | NM_002445 | 55-161  bp | F: GCAGTGGGATCACTTTCACAA R: AGCTGTCATTGAGCGAGCATC |
| *Efnb2* | human | NM_004093 | 1-820  bp | F: TATGCAGAACTGCGATTTCCAA R: TGGGTATAGTACCAGTCCTTGTC |
| *Il1a* | human | NM_000575 | 156-378  bp | F: TGGTAGTAGCAACCAACGGGA R: ACTTTGATTGAGGGCGTCATTC |
| *Efnb1* | human | NM_004429 | 1-884  bp | F: TGGAGCCCGTATCCTGGAG R: TTGGGGTCGAGAACTGTGCTA |
| *Tgfb2* | human | NM_001135599 | 1-1712  bp | F: CAGCACACTCGATATGGACCA R: CCTCGGGCTCAGGATAGTCT |
| *Fmo2* | human | NM_001460 | 61-198  bp | F: GGAGTGTGGAGGTTCAAAGAG R: TGCTGGTGTTGGTAACGACAG |
| *Prss23* | human | NM_007173 | 111-3713  bp | F: TGTGCTGTTGGGCAAGTGAG R: AGTTCCCTTATGACACTGGGG |
| *Samd5* | human | NM_001030060 | 1-698  bp | F: CACCAACATAGTTTACGAGTGGC R: TAGCCGTTATCCACGAAGGAC |
| *Bmp4* | human | NM_001202 | 402-778  bp | F: ATGATTCCTGGTAACCGAATGC R: CCCCGTCTCAGGTATCAAACT |
| *Col6a3* | human | NM_057166 | 213-333  bp | F: ATGAGGAAACATCGGCACTTG R: GGGCATGAGTTGTAGGAAAGC |
| *Gpm6a* | human | NM_005277 | 145-337  bp | F: ATTCCCTATGCCTCTCTGATTGC R: GCCATCTCAAAGTAGGTTTGCAG |
| *Wnt9b* | human | NM_003396 | 391-656  bp | F: TGTGCGGTGACAACCTCAAG R: ACAGGAGCCTGATACGCCAT |
| *Edn1* | human | NM_001168319 | 334-499  bp | F: AGAGTGTGTCTACTTCTGCCA R: CTTCCAAGTCCATACGGAACAA |
| *Fstl1* | human | NM_007085 | 265-394  bp | F: GAGCAATGCAAACCTCACAAG  R: CAGTGTCCATCGTAATCAACCTG |
| *Selp* | human | NM_003005 | 145-531  bp | F: ACTGCCAGAATCGCTACACAG R: CACCCATGTCCATGTCTTATTGT |
| *Rbms3* | human | NM_001003793 | 687-895  bp | F: GGGGAACAGTTGAGTAAAACCA  R: ACAATTTTTCCATACGGTTGGCA |
| *β-actin* | human | NM_001101 | 448-886  bp | F: CATGTACGTTGCTATCCAGGC R: GGCTGTATTCCCCTCCATCG |
| *FXR* | human | NM_001206978 | 391-545  bp | F: AGAGATGGGAATGTTGGCTGA  R: TTGTCGAGGTCACTTGTCGC |
| *PPAR-α* | human | NM_005036 | 338-498  bp | F: GGACAAGGCCTCAGGCTATC  R: CCAGGACGATCGTTGTGTGA |
| *LXR* | human | NM_007121 | 482-678  bp | F: GGGAGCAGTGCGTCCTTT  R: GCCACCAACTGCTGGATCAT |
| *TNF-α* | human | NM_000594 | 435-582  bp | F: CTCTTCAAGGGCCAAGGCTG  R: TGGAAGACCCCTCCCAGATA |
| *IL-6* | human | NM_000600 | 246-407  bp | F: AGAGGCACTGGCAGAAAACA  R: CAGCTCTGGCTTGTTCCTCA |
| *Col1α* | human | NM_000088 | 1746-1887  bp | F: CAGGCTGGTGTGATGGGATT  R: GGGCCTTGTTCACCTCTCTC |
| *α-SMA* | human | NM_001613 | 299-469  bp | F: CAATGAGCTTCGTGTTGCCC  R: GTGGGTGACACCATCTCCAG |
| *PINK1* | human | NM_032409 | 770-933  bp | F: TGGGGAGTATGGAGCAGTCA  R: ATAACGAGGAACAGCGTCCG |
| *Parkin* | human | NM_013987 | 502-586  bp | F: AACTCAGGGTACAGTGCAGC  R: TGCGATCAGGTGCAAAGCTA |
| *LC3B* | human | NM_022818 | 63-266  bp | F: CGAACAAAGAGTAGAAGATGTCCG  R: AGATTGGTGTGGAGACGCTG |
| *C-Kit* | mouse | NM_001122733 | 841-1013  bp | F: GCCAGGAGACGCTGACTATC  R: TGGGGTAGGCCTCGTATTCA |
| *ADPN* | mouse | NM_197985 | 269-304  bp | F: GGCCCATCATGCTATGGAAC  R: GTGAGGGATCACTCGCCATC |
| *PPAR-α* | mouse | NM_011144 | 34-144  bp | F: AGAGCCCCATCTGTCCTCTC  R: ACTGGTAGTCTGCAAAACCAAA |
| *FXR* | mouse | NM_001163504 | 124-143  bp | F: GGCAGAATCTGGATTTGGAATCG  R: GCTGAACTTGAGGAAACGGG |
| *LXR* | mouse | NM_013839 | 466-525  bp | F: AGGAGTGTCGACTTCGCAAA  R: CTCTTCTTGCCGCTTCAGTTT |
| *SREBP-1c* | mouse | NM_011480 | 553-711  bp | F: GCAGCCACCATCTAGCCTG  R: CAGCAGTGAGTCTGCCTTGAT |
| *TNF-α* | mouse | NM_013693 | 253-271  bp | F: CCCTCACACTCAGATCATCTTCT  R: GCTACGACGTGGGCTACAG |
| *IL-6* | mouse | NM_031168 | 511-542  bp | F: TAGTCCTTCCTACCCCAATTTCC  R: TTGGTCCTTAGCCACTCCTTC |
| *Col1α* | mouse | NM_007742 | 49-113  bp | F: GCTCCTCTTAGGGGCCACT  R: CCACGTCTCACCATTGGGG |
| *α-SMA* | mouse | NM_007392 | 137-196  bp | F: GTCCCAGACATCAGGGAGTAA  R: TCGGATACTTCAGCGTCAGGA |
| *PINK1* | mouse | NM_026880 | 956-1068  bp | F: CACACTGTTCCTCGTTATGAAGA  R: CTTGAGATCCCGATGGGCAAT |
| *P62* | mouse | NM_011018 | 228-365  bp | F: AGGATGGGGACTTGGTTGC  R: TCACAGATCACATTGGGGTGC |
| *Parkin* | mouse | NM_016694 | 767-841  bp | F: TCTTCCAGTGTAACCACCGTC  R: GGCAGGGAGTAGCCAAGTT |
| *LC3B* | mouse | NM_026160 | 124-186  bp | F: TTATAGAGCGATACAAGGGGGAG  R: CGCCGTCTGATTATCTTGATGAG |

* The used concentration of primers in the table was 10 μmol/L. The amplification efficiency of primers is between 90% and 110%.

**Table S3 Statistical analyses used in the methods**

| **Method** | **Statistical analyses** | **Analysis software** |
| --- | --- | --- |
| DEGs in scRNA-seq | Wilcoxon test and Bonferroni correction | Seurat v3.0 package |
| KEGG/GO analyses in scRNA-seq | Fisher's exact test and FDR | ClusterProfiler package |
| PCR/western blot validation | unpaired Student’s t test | SPSS 19.0 software |
| Flow cytometry | unpaired Student’s t test | SPSS 19.0 software |
| Semi-quantitative calculation of IF/IHC images | unpaired Student’s t test | SPSS 19.0 software |
| Semi-quantitative calculation of images (H&E, ORO, Masson) | unpaired Student’s t test | SPSS 19.0 software |

**Table S4 The top 10 representative DEGs of cluster 0, 1, 2 from LSECs**

| **Cluster 0** | **Ensemble ID** | ***Gene*** | **avg_logFC** | **pct of cluster 0** | **pct of other clusters** | **adj p-val** | **pct-**  **FC** |
| --- | --- | --- | --- | --- | --- | --- | --- |
| 1 | ENSMUSG00000005672 | *C-Kit* | 0.63 | 0.67 | 0.40 | 3.43E-53 | 1.68 |
| 2 | ENSMUSG00000028444 | *Cntfr* | 0.48 | 0.49 | 0.30 | 4.60E-24 | 1.61 |
| 3 | ENSMUSG00000000253 | *Gmpr* | 0.29 | 0.27 | 0.18 | 5.45E-06 | 1.53 |
| 4 | ENSMUSG00000010797 | *Wnt2* | 0.30 | 0.59 | 0.38 | 6.31E-17 | 1.53 |
| 5 | ENSMUSG00000029762 | *Akr1b8* | 0.28 | 0.31 | 0.21 | 7.12E-06 | 1.47 |
| 6 | ENSMUSG00000083929 | *Gm10600* | 0.32 | 0.25 | 0.17 | 1.41E-03 | 1.46 |
| 7 | ENSMUSG00000031129 | *Slc9a9* | 0.27 | 0.29 | 0.20 | 6.12E-04 | 1.42 |
| 8 | ENSMUSG00000021379 | *Id4* | 0.35 | 0.48 | 0.34 | 3.76E-12 | 1.40 |
| 9 | ENSMUSG00000003948 | *Mmd* | 0.37 | 0.32 | 0.23 | 1.00E-06 | 1.40 |
| 10 | ENSMUSG00000032946 | *Rasgrp2* | 0.38 | 0.43 | 0.31 | 4.35E-11 | 1.40 |
| **Cluster 1** | **Ensemble ID** | ***Gene*** | **avg_logFC** | **pct of cluster 1** | **pct of other clusters** | **adj p-val** | **pct-**  **FC** |
| 1 | ENSMUSG00000025044 | *Msr1* | 1.13 | 0.73 | 0.23 | 8.61E-135 | 3.20 |
| 2 | ENSMUSG00000001300 | *Efnb2* | 0.64 | 0.47 | 0.18 | 1.54E-48 | 2.68 |
| 3 | ENSMUSG00000027399 | *Il1a* | 0.48 | 0.34 | 0.15 | 1.27E-24 | 2.32 |
| 4 | ENSMUSG00000031217 | *Efnb1* | 0.44 | 0.41 | 0.21 | 6.53E-23 | 2.00 |
| 5 | ENSMUSG00000066363 | *Serpina3f* | 0.31 | 0.28 | 0.15 | 1.79E-09 | 1.86 |
| 6 | ENSMUSG00000019846 | *Lama4* | 0.44 | 0.44 | 0.24 | 3.44E-21 | 1.84 |
| 7 | ENSMUSG00000041361 | *Myzap* | 0.26 | 0.27 | 0.15 | 2.21E-08 | 1.83 |
| 8 | ENSMUSG00000027314 | *Dll4* | 0.46 | 0.55 | 0.31 | 1.79E-25 | 1.78 |
| 9 | ENSMUSG00000021903 | *Galnt15* | 0.32 | 0.28 | 0.17 | 2.33E-07 | 1.70 |
| 10 | ENSMUSG00000033350 | *Chst2* | 0.38 | 0.43 | 0.26 | 9.55E-14 | 1.63 |
| **Cluster 2** | **Ensemble ID** | ***Gene*** | **avg_logFC** | **pct of cluster 2** | **pct of other clusters** | **adj p-val** | **pct-**  **FC** |
| 1 | ENSMUSG00000039239 | *Tgfb2* | 0.82 | 0.30 | 0.01 | 1.53E-122 | 42.86 |
| 2 | ENSMUSG00000040170 | *Fmo2* | 1.75 | 0.32 | 0.01 | 2.20E-131 | 36.00 |
| 3 | ENSMUSG00000039405 | *Prss23* | 0.79 | 0.30 | 0.01 | 6.47E-119 | 33.33 |
| 4 | ENSMUSG00000060487 | *Samd5* | 0.729 | 0.30 | 0.01 | 4.59E-117 | 30.20 |
| 5 | ENSMUSG00000021835 | *Bmp4* | 2.08 | 0.70 | 0.03 | 5.68E-289 | 23.27 |
| 6 | ENSMUSG00000048126 | *Col6a3* | 0.63 | 0.25 | 0.01 | 7.63E-88 | 21.00 |
| 7 | ENSMUSG00000031517 | *Gpm6a* | 1.14 | 0.46 | 0.02 | 3.50E-167 | 19.87 |
| 8 | ENSMUSG00000022816 | *Fstl1* | 0.85 | 0.31 | 0.02 | 1.74E-103 | 17.22 |
| 9 | ENSMUSG00000026580 | *Selp* | 1.62 | 0.75 | 0.04 | 4.07E-283 | 16.93 |
| 10 | ENSMUSG00000039607 | *Rbms3* | 1.14 | 0.49 | 0.03 | 1.09E-164 | 15.25 |

*pct, percentage; avg_logFC, average of log fold change; adj p-val, adjust p-value; pct-FC, percentage-fold change.

**Additional Figure Legends**

**Figure S1 Histological examination of mice model**

Representative images of H&E staining (A), Masson (B), ORO (C) and IHC of F4/80 staining (D) of livers from control mice (n=3) and MCD fed mice (n=3). NASH activity scores including steatosis, inflammation and ballooning (E), Masson (F), ORO (G) and IHC F4/80 (H) in liver sections were calculated between 2 groups. Scale bar=100 μm in (A-C); Scale bar=200 μm in (D).

Data were showed as the mean ± SEM. The *p-*values were calculated using an unpaired Student’s t test. *p*-value means the statistical significance compared to control (Con) mice

**Figure S2** **Expression of 6 DEGs of cluster 0**

Paired tSNE plots (left) and violin plots (right) of 6 DEGs among the top 10 DEGs: (A) *Akr1b8*, (B) *Gm10600*, (C) *Slc9a9*, (D) *Id4*, (E) *Mmd* and (F) *Rasgrp2*.

**Figure S3** **Expression of 6 DEGs of cluster 1**

Paired tSNE plots (left) and violin plots (right) of 6 DEGs among the top 10 DEGs: (A) *Serpina3f*, (B) *Lama4*, (C) *Myzap*, (D) *Dll4*, (E) *Galnt15* and (F) *Chst2*.

**Figure S4 Expression of 5 DEGs of cluster 2**

Paired tSNE plots (left) and violin plots (right) of 5 DEGs among the top 10 DEGs: (A) *Tgfb2*, (B) *Fmo2*, (C) *Prss23*, (D) *Fstl1* and (E) *Rbms3*.

**
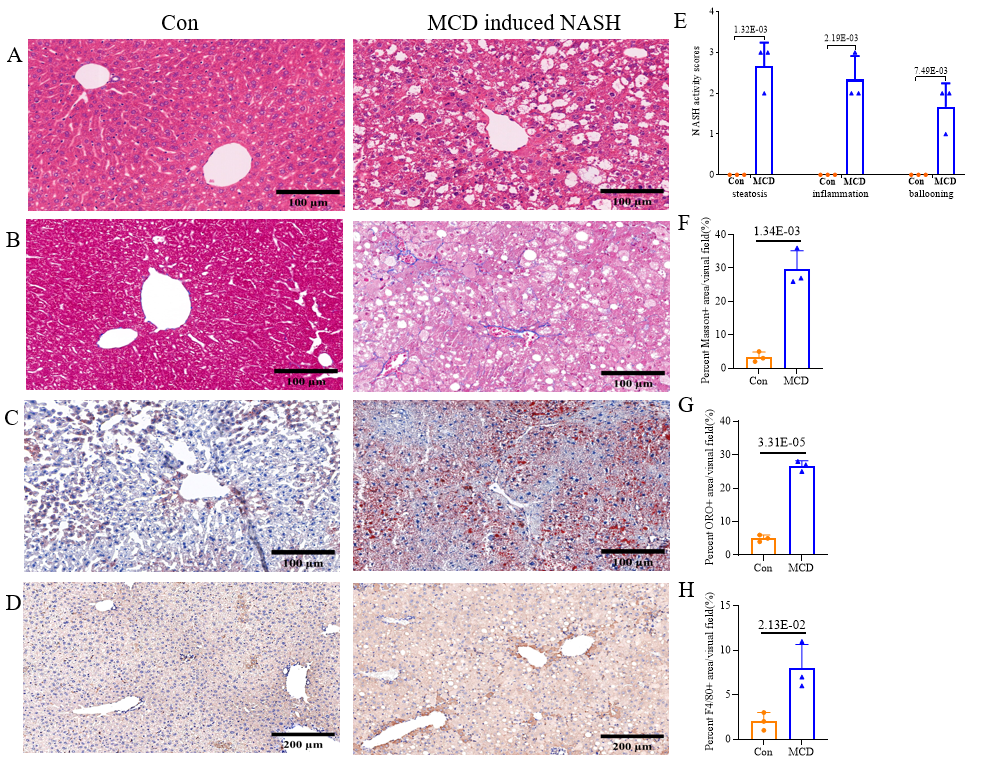
**

**
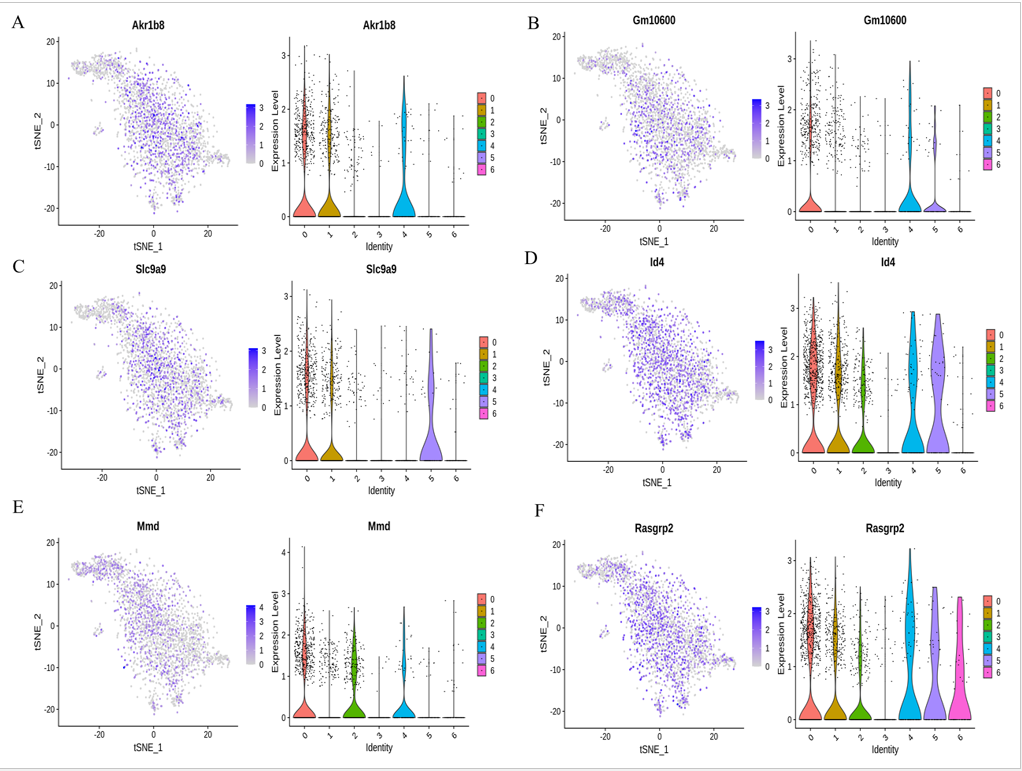
**

**
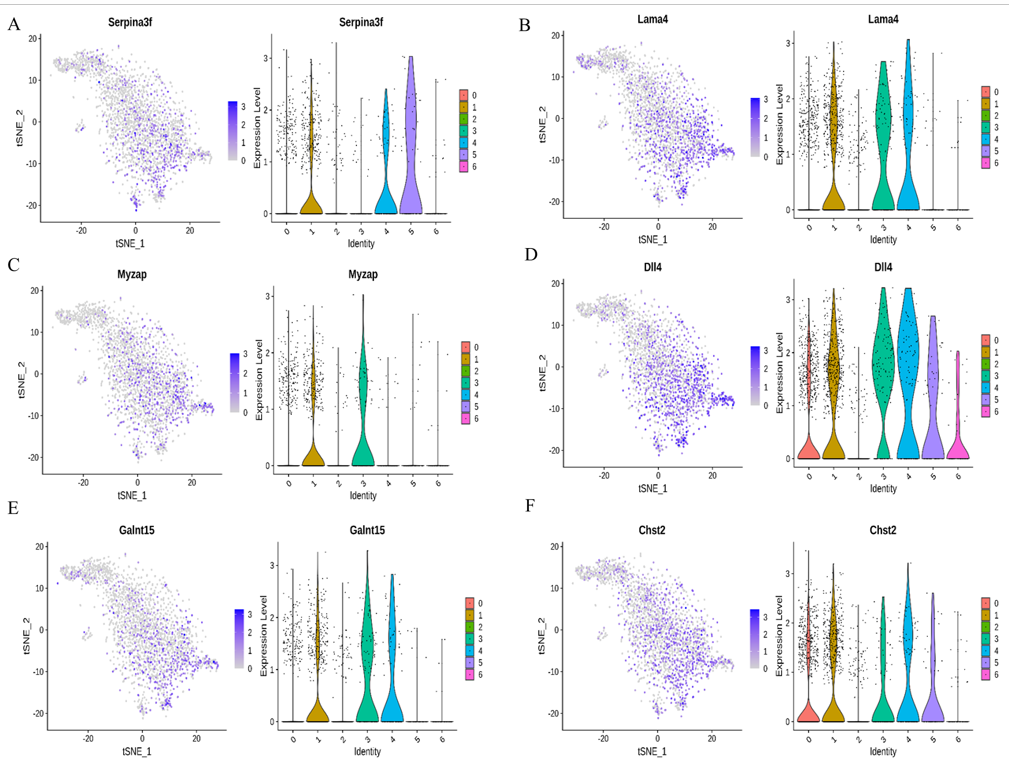
**

**
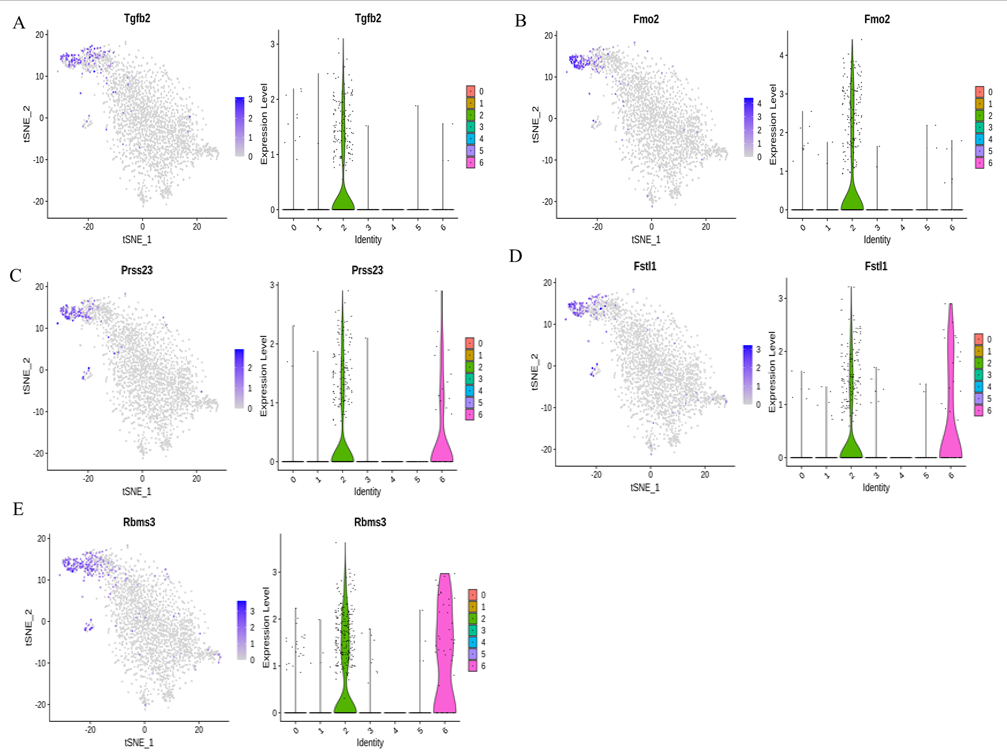
**
